# Long-term Video-EEG Monitoring Findings in Children and Adolescents with Intractable Epilepsy

**Published:** 2017

**Authors:** Yasaman GHAZAVI, Ebrahim Asayesh ZARCHI, Taher TAHERI, Mahdi SAFIABADI, Elham RAHIMIAN, Susan AMIRSALARI

**Affiliations:** 1 Shefa neuroscience research Center, Rashid Yasemi ST,Tehran, Iran.

**Keywords:** Long term video-EEG monitoring (LTM), intractableepilepsy, Epilepsy surgery

## Abstract

**Objective:**

Long Term Video-EEG Monitoring (LTM) may give us important information in the preoperative assessment of these patients. We performed this study for the first time in pediatric age group in Iran.

**Materials and Methods:**

In this cross-sectional study, 43 children between 4 to 18 yr, with intractable epilepsy referred to Shefa Neuroscience Research Center, Tehran, Iranfrom2007-2012, were enrolled to study in order to evaluate their long-term video EEG findings.

**Results:**

The patients mean age was10.07 yr, from which 24(65.9%) were boys.Seven patients with definite epileptogenic zone were advised to perform lesionectomy surgery.In two patients, there was not any seizure onset focus but corpus callosotomy was advised to control their frequent falling.Eight cases were recommended to perform electrocorticography or invasive EEG monitoring and26 cases to adjust medical treatment. In three cases, there was not any electrical seizure activity during clinical attacks, so discontinuing anti-epileptic drugs were recommended fordiagnosis of conditions that mimic epilepsy.

**Conclusion:**

It is necessary to perform LTM in patients with refractory epilepsy in order to determine their treatment strategy. If there is any doubt about pseudoseizureLTM can help to differentiate epilepsy from conditions that mimic epilepsy.

## Introduction

A seizure is a paroxysmal involuntary, time-limited change in brain function, resulting from abnormal discharges from cerebral neurons. It is called epilepsy if it occurs two times or more without any provocation (1-3). Intractable epilepsy, if untreated, can lead to cognitive decline, impaired mental and social status, lifestyle disruption and patient dependency (4). The biologic basis of seizure recurrence lies in conditions such as severe epileptic syndromes, underlying neuropathological diseases, abnormal reorganization of neurons, replacement of receptors and neurotransmitters, ion channel disorders, reactive autoimmune disorders, and improper use of anti-epileptic drugs (4). Epileptic seizures may occur due to exposure to bright lights, video games, repetitive sounds, sleep deprivation, excessive alcohol consumption, stress, smoking and hormonal changes (5). 

The annual incidence of epilepsy is 0.5%-0.8%and cumulative incidence is about 3% during lifetime; more than half of the cases occur in childhood (1). A seizure is resolved in 60%-70% of children with epilepsy after a year or two with medications. However, its attacks continue in 10%-20% of children despite receiving appropriate medications (2). Failure in initial treatment with antiepileptic drugs may be due to inappropriate drugs dosages, ineffectiveness of the type medications, or drug intolerance (4).

Medical treatment is effective and the primary method of epilepsy treatment depends on a number of factors including patient's age, type of epilepsy, drug interactions, ease of use and side effects of medications. 

Depending on the type and number of seizures, patient’s condition or the underlying cause, some non-pharmacologic treatments such as brain surgery, vagus nerve stimulation, or ketogenic diet might be used in specific cases, where patient does not respond to medical treatment and his or her normal life is disrupted (6).

**Fig 1:**
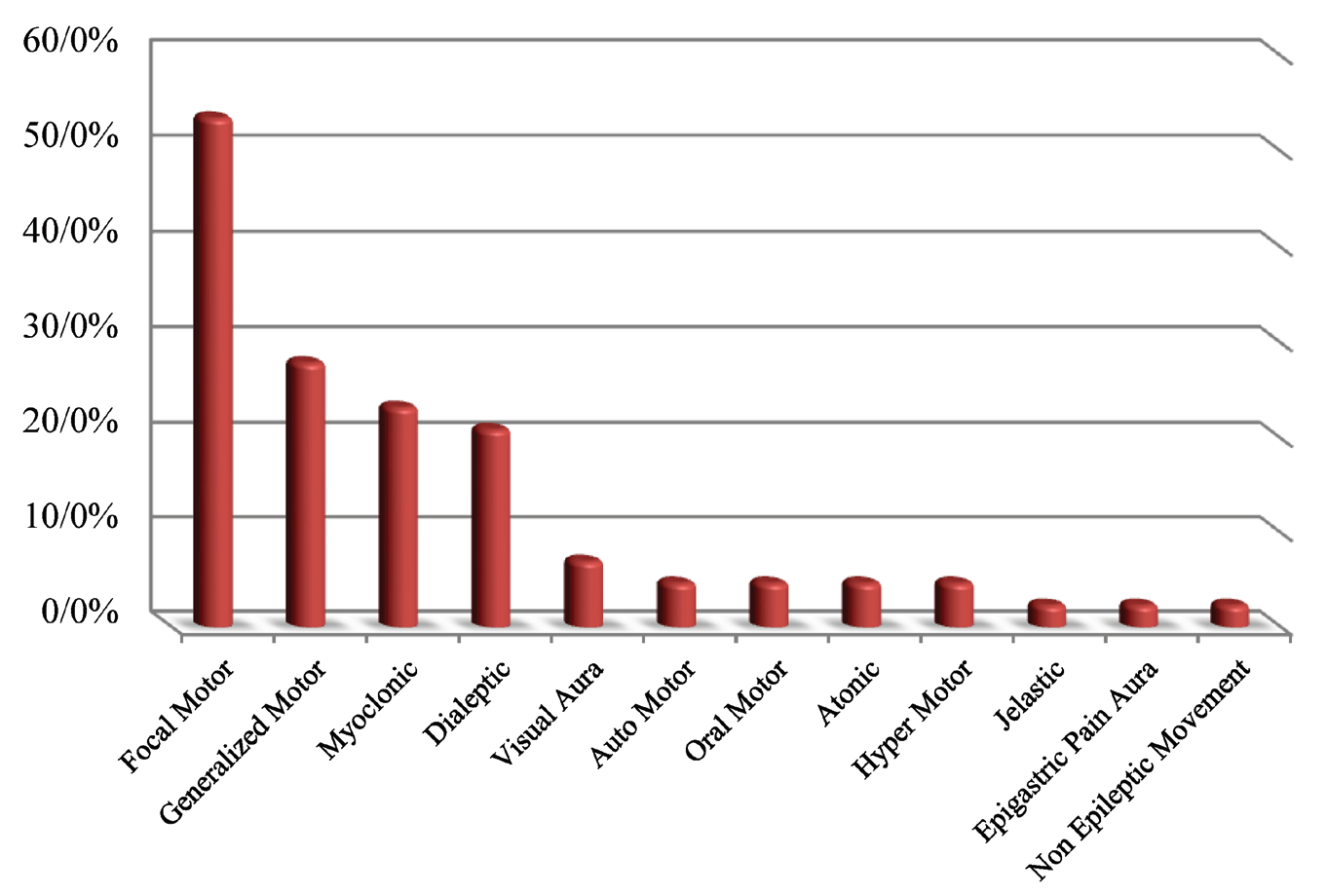
Semiology of the clinical attacks in patients

**Table1:** Relationship between age and the type of seizure

Type of seizure	Patients	Age Average	Std. Deviation
Partial	24	9.75	4.54
Generalized	11	10.73	3.77
Mixed	8	10.13	3.27
Total	43	10.07	3.83
*P*	0.790		

**Table 2:** Relationship between Ictal EEG and type of seizure and epileptogenic zone

	Normal Ictal EEG N(%)	Abnormal Ictal EEG N(%)	Total N(%)	*P*
Generalized seizures	(0) 0	(9.28) 11	(6.25) 11	359.0
Partial seizures	(80) 4	(6.52) 20	(4.55) 24	
Mixed type seizures	(20) 1	(5.18) 7	(6.18) 8	
Detected epileptogenic zone	(20) 1	(50) 19	(5.46) 20	210.0
Undetected epileptogenic zone	(80) 4 (50) 19 (5.53) 23			

**Table 3:** Relationship between Interictal EEG and type of seizure and epileptogenic zone

		Normal Interictal EEG/ N (%)	Abnormal Interictal EEG N (%)	Total N (%)	*P*
	Generalized seizures	(0) 0	(2.28) 11	(6.25) 11	083.0
	Partial seizures	(100) 4	(3.51) 20	(4.55) 24	
	Mixed type seizures	(0) 0	(5.20) 8	(6.18) 8	
	Detected Epileptogenic zone	(25) 1	(7.48) 19	(5.46) 20	350.0
	Undetected Epileptogenic zone	(75) 3	(3.51) 20	(5.53) 23	

**Table 4:** Relationship between EEG findings and the number of seizure attacks

	EEG findings	Patients	Number of attacks (mean ±SD)	*P*
Ictal EEG	Normal	5	(19.47±)39	610.0
	Abnormal	38	(37.37±)74.29	
Interictal EEG	Normal	4	(03.74±)25.65	047.0
	Abnormal	39	(16.32±)28.27	

**Table 5:** Relationship between the EEG findings and Brain MRI

		Normal Brain MRI	Abnormal Brain MRI	Total	*P*
Ictal EEG	Normal	2	3	5	0.579
	Abnormal	13	25	38	
Inter Ictal EEG	Normal	2	2	4	0.436
	Abnormal	13	26	39	

**Table 6.** Relationship between the EEG findings, Brain MRI findings and the type of recommended treatment

		Surgical treatment	Medical treatment	Electrocorticography or Invasive EEG Monitoring	*P*
Brain MRI	Normal	0 (0)	14 (93.3)	1 (6.7)	0.005
	Abnormal	9 (32.1)	12 (42.9)	7 (25)	
Ictal EEG	Normal	0 (0)	5 (100)	0 (0)	0.065
	Abnormal	9 (23.6)	21 (55.4)	8 (21)	
Inter Ictal EEG	Normal	0 (0)	3 (75)	1 (25)	0.550
	Abnormal	9 (23.1)	21 (53.9)	8 (23)	

**Table7:** Relationship between recommended treatment and results of LTM

Types of treatment	Patients N (%)	Epileptogenic zone N (%)	
Surgical treatment	(9.20) 9	Detected	(7.77) 7
		Undetected	(3.22) 2
Invasive EEG Monitoring or Electrocorticography	(7.19) 8	Detected	25) 2)
		Undetected	75) 6)
Medical treatment	(4.60) 26	Detected	(3.42) 11
		Undetected	(7.57) 15

**Table 8.** Summary of Demographic, Electroencephalographic, Neuroimaging findings and final recommendations in 43 cases undergone LTM

	Sex	Age	MRI Finding	Inter Ictal EEG	Ictal EEG	Recommendation
1	Female	12	Bilateral polymicrogyria and periventricular heterotopia	Nonepileptiform: generalized epileptiform: anterior region of head	Generalized	Surgical
2	Male	16	Old hydrocephaly	Bilateral	Nonepileptiform: bilateral epileptiform: left posterior	Continue AEDs
3	Male	9	Global atrophy of the right hemisphere, Gliosis of right occipital	Left hemisphere	Epileptiform: bilateral	Continue AEDs
4	Female	12	Left partial focal dysplasia	Left hemisphere	Epileptiform: Left side	Surgical
5	Male	17	Signal abnormality in right frontotemporal (growing glioma)	Right fronto temporal	Nonepileptiform:right Frontotemporal Epileptiform: right frontotemporal	Surgical
6	Female	6	Global atrophy- left hippocampal atrophy	Bilateral	Epileptiform: right hemisphere	Continue AEDs
7	Female	6	Abnormal right hippocampal atrophy	NORMAL	NORMAL	Continue AEDs
8	Male	9	NORMAL	Bifrontal	Non epileptiform: bilateral, Epileptiform: bilateral	Continue AEDs
9	Male	8	NORMAL	NORMAL	NORMAL	Discontinue AEDs
10	Male	8	Pachygyria lesion over the right frontal	Bilateral fronto temporal	Right anterior temporal	Invasive monitoring
11	Female	8	Lesion on left posterior frontal	Left side	Left central area	Continue AEDs
12	Male	9	NORMAL	Right hemisphere	Right temporal	Continue AEDs
13	Male	4	Widening in frontotemporal (porencephalic cyst)	Bilateral	Bilateral	Surgical
14	Male	4	NORMAL	Frontocentral area	Left posterior head	Continue AEDs
15	Female	5	Abnormal gyration in both frontal	Multi focal	Bifrontal	Continue AEDs
16	Female	13	Hypogenesis of corpus callosum	Right Frontocentral	NORMAL	Invasive monitoring
17	Male	9	Cerebellar atrophy	Generalized	Multi focal	Continue AEDs
18	Male	8	Left sided hippocampal atrophy	Left hemisphere	Left hemisphere	Continue AEDs
19	Female	7	Left hippocampal sclerosis	Left posterior temporal & Right anterior temporal	Both hemisphere	Invasive monitoring
20	Male	8	Increasing signal in hippocampuses ( hipocampal sclerosis) volume loss in both hemisphere (atrophy)	NORMAL	Bilateral parieto central	Continue AEDs
21	Female	4	NORMAL	Bilateral	Generalized	Continue AEDs
22	Male	15	Abnormal signal in left parietal lobe cortex	Left temporal	Left temporal	Surgical
23	Female	5	Mass lesion in inferior thalamus	Left hemisphere	Non epileptiform on left hemisphere	Invasive monitoring
24	Female	10	NORMAL	NORMAL	NORMAL	
25	Male	10	NORMAL	Generalized	Generalized	Continue AEDs
26	Female	7	Atrophy and porencephalic cyst on left hemisphere	Left hemisphere	Left hemisphere	Invasive monitoring Discontinue AEDs
27	Female	8	Bilateral hippocampal atrophy	NORMAL	Bilateral posterior temporal	Continue AEDs
28	Female	6	Bilateral cortical malformation	Left posterior head	Left posterior head	Surgical
29	Female	13	NORMAL	Bilateral	Epileptiform: generalized	Continue AEDs
30	Female	10	Mild bilateral hippocampal roundening and medial displacement	Generalized	Epileptiform: bilateral	Continue AEDs
31	Male	9	NORMAL	Left frontal	Bifrontal, maximum left frontal	Continue AEDs
32	Female	8	NORMAL	Frontal	Multi focal maximum frontocentral	Continue AEDs
33	Male	17	NORMAL	Generalized	Multifocal	Continue AEDs
34	Male	8	Volume loss in right lobe and dilatation on temporal horn of right lateral ventricle	Right temporal	Bifrontotemporal, maximum right temporal	Surgical
35	Male	11	Volume loss in right hippocampal and right sided hippocampal sclerosis	Right temporal	Non epileptiform, bilateral	Surgical
36	Female	13	NORMAL	Right frontal & left hemisphere	Bilateral frontotemporal	Continue AEDs
37	Male	17	Signal change in right occipital	Right posterior head	Posterior head	Continue AEDs
38	Male	14	Global atrophy	Right temporal	Right hemisphere	Surgical
39	Male	12	Parietocentral lesion	Left central and right hemisphere	Multifocus	Invasive monitoring
40	Female	12	NORMAL	Bilateral	Right centropariental	Invasive monitoring
41	Male	16	Brain malacia with gliosis and atrophy in left fronto temporoparietal, mild patchy gliosis in right parieto-occipital	Started bilateral ,evolutionright frontal	Right frontal	Invasive monitoring
42	Male	16	NORMAL	Generalized	Multifocal	Continue AEDs
43	Female	14	NORMAL	Bilateral	Bilateral	Continue AEDs

Despite similar seizure disorders or similar lesions on MRI, patients with epilepsy respond differently to treatment and some are resistant or refractory to all available treatments (1). A refractory seizure or refractory epilepsy is defined as seizure activity, which occurs at least once a month for at least two years, despite treatment with three antiepileptic drugs (7). 

Patients’ medical history, seizure semiology, and electroencephalogram (EEG) are usually used for diagnosis. Long-term electroencephalography or video-EEG help to identify abnormal brain waves (6). In some patients with refractory epilepsy, the focus of seizure can be diagnosed by brain CT scan or MRI. However, in some patients, there are microscopic abnormalities diagnosed by Long-term Video-EEG Monitoring (LTM) (8). LTM is a specialized form of EEG performed by continuous monitoring of brain activity and video recording of clinical behavior. To perform the LTM, the patient will be hospitalized and his brain waves and clinical images will be recorded for 12 h to a few days. Hence, clinical symptoms and brain waves can be studied simultaneously (12). The test is noninvasive and patient feels no pain or discomfort during hospitalization. The test allows the physician to review the patient's brain electrical activity when he/she has abnormal behaviors or seizure attacks; to determine the focus of seizure in the brain, diagnose the nature of invasive abnormalities, and select the best and most effective method of treatment (medical or surgical) (8). LTM has been very effective in patients with frequent attacks whose definite diagnosis could not be reached by conventional methods. Using LTM in patients with epilepsy, physician can monitor patient’s 24 h activities, both during sleep and waking hours;to determine the type and frequency of seizures (8). Furthermore, this test helps physician to determine the focus of seizure, differentiate between nonepileptic and epileptic seizures, classify attacks, detect epileptic syndromes, determine the number of seizures and epilepsy mimicking disorders (i.e. tic disorders, sleepwalking disorders, night terrors and cataplexy) (8, 9). Analyzing the results of LTM during and between attacks provides precise knowledge of brain points’ function (10, 11). 

As LTM is a new method and due to the lack of studies in this field in Iran, for the first time in Iran, this study aimed to evaluate the long-term video-EEG findings in pediatric and young patients of 4 to 18 yr with refractory epilepsy. 

## Materials and Methods

This cross-sectional study was conducted from 2007 to 2012, on all pediatric and young patients of 4 to 18 yr referred to Shefa Neuroscience Research Center, Tehran, Iran. They were diagnosed with refractory epilepsy. Inclusion criteria included having a medical diagnosis of refractory epilepsy made by a specialist in Pediatric Neurology and not having a history of surgery for epilepsy.Using a consecutive sampling method, all the 43 patients with refractory epilepsy were recruited in the study. 

Ethics permissions for the study were obtained from the hospital authorities and an informed consent was signed by parents of patients. 

Data collection instrument consisted of two parts. The first part included questions on the patient’s age, sex, date of the first seizure, the number of seizures in 2 yr, drugs used so far (three drugs or less) and type of seizure (generalized, partial, mixed). The second part of the data collection instrument included items on EEG findings during the attack (ictal EEG), EEG findings between attacks (interictal EEG), location of the seizure focus, brain MRI information, and the LTM data. The needed data were gathered from the patients’ hospital flies or through clinical observations and interviews with parents. 

LTM and behavioral observationswere performed on all patients for several hours to several days. In each case, the LTM was stopped and the results were recorded after two or more clinical seizures. 

Statistical analysis was carried out using SPSS software version 17(Chicago, IL, USA).Kolmogorov-Smirnov test was performed to examine the normality of the data. Then, Mann-Whitney U, analysis of variance (ANOVA) and Fisher's exact tests were used to investigate the relationship between variables. Statistical significance was considered at *P*-value <0.05. 

## Results

Totally, 43 patients including 24 boys (55.82%) and 19 girls (44.18%) were enrolled in this study. The mean age of boys and girls was8.7 ± 12 and 11.13 ± 8 yr, respectively.Twenty-four people (55.82 %) had partial seizures, while 11 (25.58%) and eightcases(18.6%) had generalized or mixed type seizures, respectively. No significant relationship was found between patients’ age and type of seizure (*P*=0.790) (Table 1). 

Regarding theclinical semiology of seizure attacks, 53.4% (n= 23) of attacks were focal motor, followed by generalized motor (n= 12, 27.9%), myoclonic (n= 10, 23.2%), dialeptic (n= 9, 20.9%), visual aura (n=3, 6.9%), auto motor (n= 2, 4.6%), oral motor (n= 2, 4.6%), atonic (n= 2, 4.6%), hyper motor (n= 2, 4.6%), jelastic (n= 1, 2.3%), epigastric pain aura (n= 1, 2.3%), and non-epileptic attacks (n= 1, 2.3%)(Figure1).

Findings of electroencephalography and Brain MRI were as follows:

Five patients (11.63%) had normal Ictal EEG while 38 cases(88.37%) had abnormal Ictal EEG. Table 2 shows the relationship between Ictal EEG and seizure type and epileptogenic zone.

Moreover, four patients (9.30%) had normal Interictal EEG and 39 patients (90.69%) had abnormal interictal EEG. Table 3 shows the relationship between interictal EEG and seizure type and epileptogenic zone.

There wasno significant relationship between seizure type and focus and EEG findings(Table 2 and 3).

There wassignificant relationship between type and number of attacks (*P*=0.047) (Table 4). Moreover, no significant relationship was found between ictal and interictal EEG findings and brain MRI findings (*P*=0.579 and *P*=0.436) (Table 5). However, a significant relationship was found between resultsof brain MRI and the type of treatment (*P*=0.005). Nonetheless, nosignificant relationship was found between ictal and interictal EEG findings and the type of treatment (Table 6). 

Table 7 shows the final medical recommendations prescribed after reviewing the results of LTM and the dedicated brain MRI in 43 patients. First group: Nine patients were recommended having surgery; seven patients (77.7%) had a localized seizure focus were recommended to have lesionectomy surgery. Two patients (22.3%) had no seizure focus but they were recommended to have Corpus callosotomy surgery to prevent frequent falling due to frequent seizures. 

Second group: Eight patients were recommended electrocorticography or invasive EEG monitoring to determine the seizure focus precisely. 

Third group: 26 patients were recommended to continue but reform medical treatments. Seizure focuses of two patients (8%) were well defined but they were recommended to continue pharmacological treatment because it was at frontotemporal area and there were high risks for motor cortical damaged during surgery. Attacks of three patients were seizure imitators (11.5%), therefore they were recommended to discontinue treatment. One of these patients had hemifacial spasms, one had autism spectrum disorder and one had a non-epileptic attack recommended seeking psychiatric consultation (Table 8).

## Discussion

LTM is a specialized form of brain EEG done as continuous and long-term monitoring of brain activity and video recording of clinical behavior. It leads physicians to select the best and most effective way of treatment for patients with refractory epilepsy (8). Using LTM and Brain MRI we could correctly diagnose the focus of epilepsy in 20 (46.51%) of 43 pediatric patients with refractory epilepsy.

Using LTM, 60 patients were studied with 10 yr of epilepsy. Then, 40% of patients were diagnosed as having pseudoseizures. Therefore, they strongly recommended that LTM is used in diagnosis of symptomatic seizures (13). The effect of LTM was examined before surgery in 56patients with refractory epilepsy. LTM can detect about 90% of seizure types (9). In this study, the power of LTM in diagnosing the type of seizure was confirmed and 55.8% of seizures were partial and 25.5% were generalized while 6.9% of patients had pseudoseizures. The low incidence of pseudoseizures in pediatric group seems reasonable.

In this study, 6.9% of patients were diagnosed as pseudoseizures and were referred to discontinue drugs. Moreover, eight patients (19.7%) were referred for invasive monitoring. In a study, 454 patients in age range of 11 d to 20 yr were studied using LTM. Totally, 23.6%and 24.9%of patients were diagnosed as generalized or partial seizures respectively; while 35% had pseudoseizures that were recommended to discontinue antiepileptic drugs and nine cases (2%) were referred for invasive monitoring (14). 

In the current study, among three patients (6.9%) with pseudo seizures, one patient consumed more than three antiepileptic drugs and two cases consumed three antiepileptic drugs. In a study, 33 (18%) out of 182 patients with refractory epilepsy had pseudo seizures and consumed more than 1.5 antiepileptic and 1.5 psychiatric medications (15). 

**In Conclusion, **as a non-invasive diagnostic method, LTM is very useful not only in diagnosing and differentiating the type of seizures (false or true); however, in localizing the seizure focus in children with refractory epilepsy. Therefore, facilities for LTM are created at all specialized centers of epilepsy. Then, the quality of diagnosis and treatment of refractory epilepsy and consequently the quality of life of patients would be improved. 

## References

[1] French JA (2007). Refractory epilepsy: clinical overview. Epilepsia.

[2] Berg AT, Kelly MM (2006). Defining intractability: comparisons among published definitions. Epilepsia.

[3] Angus-Leppan H, Parsons LM Epilepsy: epidemiology, classification and natural history. Medicine.

[4] Kwan P, Brodie MJ (2002). Refractory epilepsy: a progressive, intractable but preventable condition?. Seizure.

[5] Fenwick P, Blumer D, Caplan R, Engel Jr (1993). Presurgical psychiatric assessment. Surgical Treatment of the Epilepsies.

[6] Elshoff L, Groening K, Grouiller F, Wiegand G, Wolff S, Michel C (2012). The value of EEG-fMRI and EEG source analysis in the presurgical setupof children with refractory focal epilepsy. Epilepsia.

[7] Lagae L, Buyse G, Deconinck A, Ceulemans B (2003). Effect of levetiracetam in refractory childhood epilepsy syndromes. Eur J Paediatr Neurol : EJPN : Eur J Paediatr Neurol.

[8] Sainju RK, Wolf BJ, Bonilha L, Martz G (2012). Relationship of number of seizures recorded on video-EEG to surgical outcome in refractory medial temporal lobe epilepsy. Arq Neuropsiquiatr.

[9] Zhou Q, Hou X, Huang Z, Wang G (2012). Slow anti-epileptic drug taper protocol in video-EEG monitoring for presurgical evaluation of epilepsy [Chinese Language]. Nan Fang Yi Ke Da Xue Xue Bao.

[10] Holmes GL, Sackellares JC, McKiernan J, Ragland M, Dreifuss FE Evaluation of childhood pseudoseizures using EEG telemetry and video tape monitoring. J Pediatr.

[11] Ashtari F, Zare M, Akrami S (2011). Clinical and Paraclinical Findings in Admitted Patients in Epilepsy Ward. IUMS.

[12] Yu HJ, Lee CG, Nam SH, Lee J, Lee M (2013). Clinical and ictal characteristics of infantile seizures: EEG correlation via long-term video EEG monitoring. Brain Dev.

[13] Duchowny MS, Resnick TJ, Deray MJ, Alvarez LA Video EEG diagnosis of repetitive behavior in early childhood and its relationship to seizures. Pediatr Neurol.

[14] Arrington DK, Ng YT, Troester MM, Kerrigan JF, Chapman KE (2013). Utility and safety of prolonged video-EEG monitoring in a tertiary pediatric epilepsy monitoring unit. Epilepsy Behav.

[15] Aghaee Hakak M, Amiri H, Mohammadpour M, Vosough I, Razavi B, Ashraf H (2013). Diagnostic and Therapeutic Role of Long Term Video-EEG Monitoring in Patients With Psychogenic Non-Epileptic Attacks. Razavi Int J Med.

